# Targeting HMGB1: A Potential Therapeutic Strategy for Chronic Kidney Disease

**DOI:** 10.7150/ijbs.87964

**Published:** 2023-09-25

**Authors:** Tongtong Liu, Qian Li, Qi Jin, Liping Yang, Huimin Mao, Peng Qu, Jing Guo, Bo Zhang, Fang Ma, Yuyang Wang, Liang Peng, Ping Li, Yongli Zhan

**Affiliations:** 1 Guang'anmen Hospital, China Academy of Chinese Medical Sciences, Beijing, China.; 2 China-Japan Friendship Hospital, Institute of Medical Science, Beijing, China.; 3 Institute of Basic Research in Clinical Medicine, China Academy of Chinese Medical Sciences, Beijing, China.

**Keywords:** HMGB1, CKD, renal homeostasis, AKI-to-CKD transition, therapeutic strategy

## Abstract

High-mobility group protein box 1 (HMGB1) is a member of a highly conserved high-mobility group protein present in all cell types. HMGB1 plays multiple roles both inside and outside the cell, depending on its subcellular localization, context, and post-translational modifications. HMGB1 is also associated with the progression of various diseases. Particularly, HMGB1 plays a critical role in CKD progression and prognosis. HMGB1 participates in multiple key events in CKD progression by activating downstream signals, including renal inflammation, the onset of persistent fibrosis, renal aging, AKI-to-CKD transition, and important cardiovascular complications. More importantly, HMGB1 plays a distinct role in the chronic pathophysiology of kidney disease, which differs from that in acute lesions. This review describes the regulatory role of HMGB1 in renal homeostasis and summarizes how HMGB1 affects CKD progression and prognosis. Finally, some promising therapeutic strategies for the targeted inhibition of HMGB1 in improving CKD are summarized. Although the application of HMGB1 as a therapeutic target in CKD faces some challenges, a more in-depth understanding of the intracellular and extracellular regulatory mechanisms of HMGB1 that underly the occurrence and progression of CKD might render HMGB1 an attractive therapeutic target for CKD.

## 1. Introduction

Chronic kidney disease (CKD), a devastating disease affecting human health worldwide, is characterized by progressive and irreversible nephron loss, reduced renal regenerative capacity, microvascular damage, changes in inflammation, metabolic and oxidative stress, and fibrosis, ultimately leading to renal failure and end-stage renal disease (ESRD)^1^, ^2^. CKD affects approximately 10-14% of the global population and is the leading cause of ESRD and premature death^3^. The contribution of CKD to global mortality is rapidly increasing due to the rising prevalence of diabetes, hypertension, obesity, and an aging population^4^. However, current treatments have limited efficacy and merely delay disease progression. Therefore, it is essential to identify new potential therapeutic targets to halt or reverse CKD progression.

**Figure 1:**
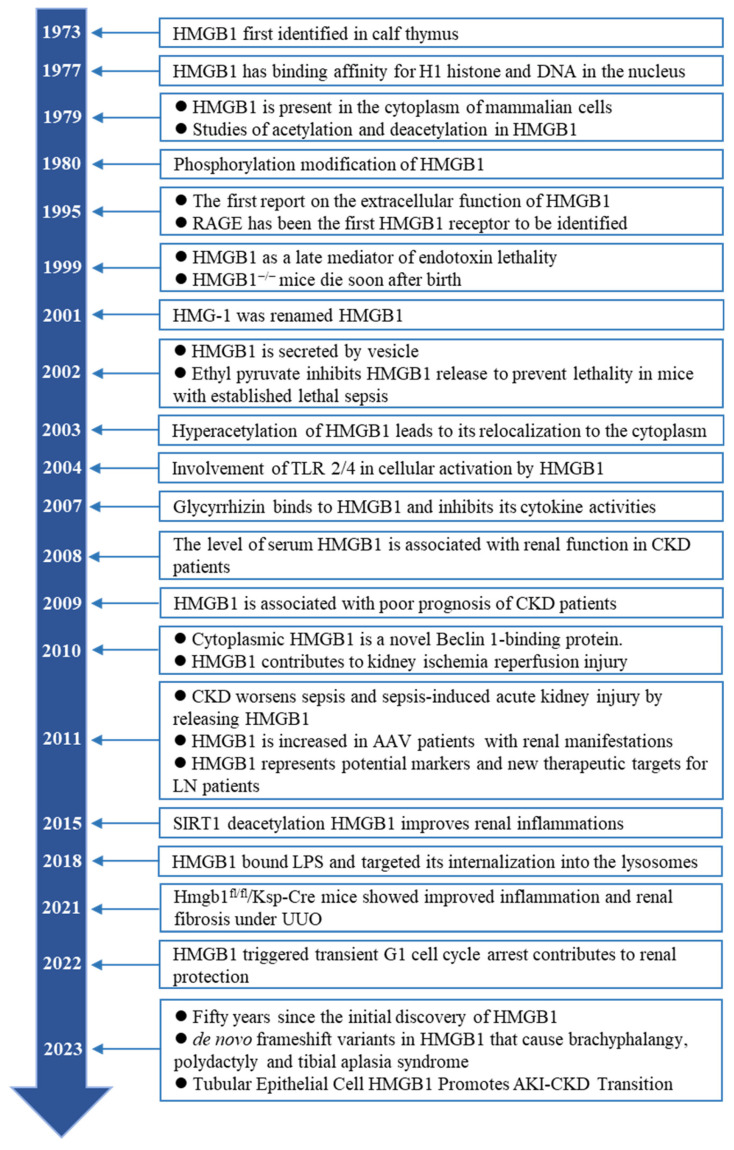
Timeline of landmark achievements of HMGB1 in CKD in the past 50 years.

High-mobility group protein box 1 (HMGB1) is a member of the high-mobility group proteins with secretory and intracellular activities^5^. HMGB1 is ubiquitously expressed in almost all cell types and is involved in cellular damage and repair. The biological activity of HMGB1 depends on its subcellular localization, context, and post-translational modifications (PTMs). HMGB1 acts as a DNA chaperone in the nucleus and is involved in DNA repair, chromatin remodeling, nucleosome assembly, and telomere maintenance. In the cytoplasm, HMGB1 acts as an autophagy maintainer and mitochondrial homeostasis regulator to regulate cell death. Extracellularly, HMGB1 acts as a damage-associated molecular pattern (DAMPs) or alarmin to activate the immune response and promote cell migration and proliferation^6^. HMGB1 has been discovered for 50 years (Figure 1). In recent years, an increasing number of HMGB1 inhibitors have shown promising therapeutic potential for a variety of diseases^7^, ^8^. HMGB1 plays an indispensable role in the pathogenesis and progression of CKD. The kidney is the best responder to HMGB1 because of the largest changes in HMGB1 in kidney tissue in the early stage of hemorrhagic shock^9^. Under CKD conditions, HMGB1 is elevated in the plasma, serum, and urine and is closely related to the progression and prognosis of CKD. As a core player, activated HMGB1 participates in multiple key events of CKD progression through the activation of downstream signals, including renal inflammation, the development of persistent fibrosis, renal aging, AKI-CKD transition, and important cardiovascular complications. Interestingly, studies have shown that HMGB1 deletion in renal tubules has no noticeable effect on renal injury in the early stage after unilateral ureteral obstruction (UUO) but greatly alleviates renal interstitial fibrosis in the late/subacute stage^10^, suggesting that HMGB1 may play additional roles in CKD than differ from those in acute kidney injury.

In this review, we evaluate the links between HMGB1 and CKD, starting with a description of the biological characteristics of HMGB1 in CKD and its regulatory role in renal homeostasis, followed by valuable preclinical and clinical evidence, summarizing how HMGB1 regulates key events related to CKD to affect its progression and prognosis. Finally, we describe strategies to reduce or inhibit HMGB1 in CKD, providing insights into the innovation of therapeutic strategies targeting HMGB1 in CKD.

## 2. The biology of HMGB1

The HMGB protein family is the most abundant among high-mobility groups. Four members (HMGB1, HMGB2, HMGB3 and HMGB4) of the mammalian HMGB family have been identified so far; among them, HMGB1 shows the highest expression. HMGB1, a non-histone nuclear protein, was first discovered in 1973 and named for its high electrophoretic mobility. HMGB1 is highly evolutionarily conserved, as evidenced by the 99% homology between rodent and human amino acid sequences. HMGB1 is essential for life as mice with systemic HMGB1 deletions die from hypoglycemia shortly after birth^11^. Of note, HMGB1 can cross organelles from the nucleus at higher concentrations into the cytoplasm in response to stress injury within 1-2 seconds^12^.

### 2.1 The structure and distribution of HMGB1

**Figure 2:**
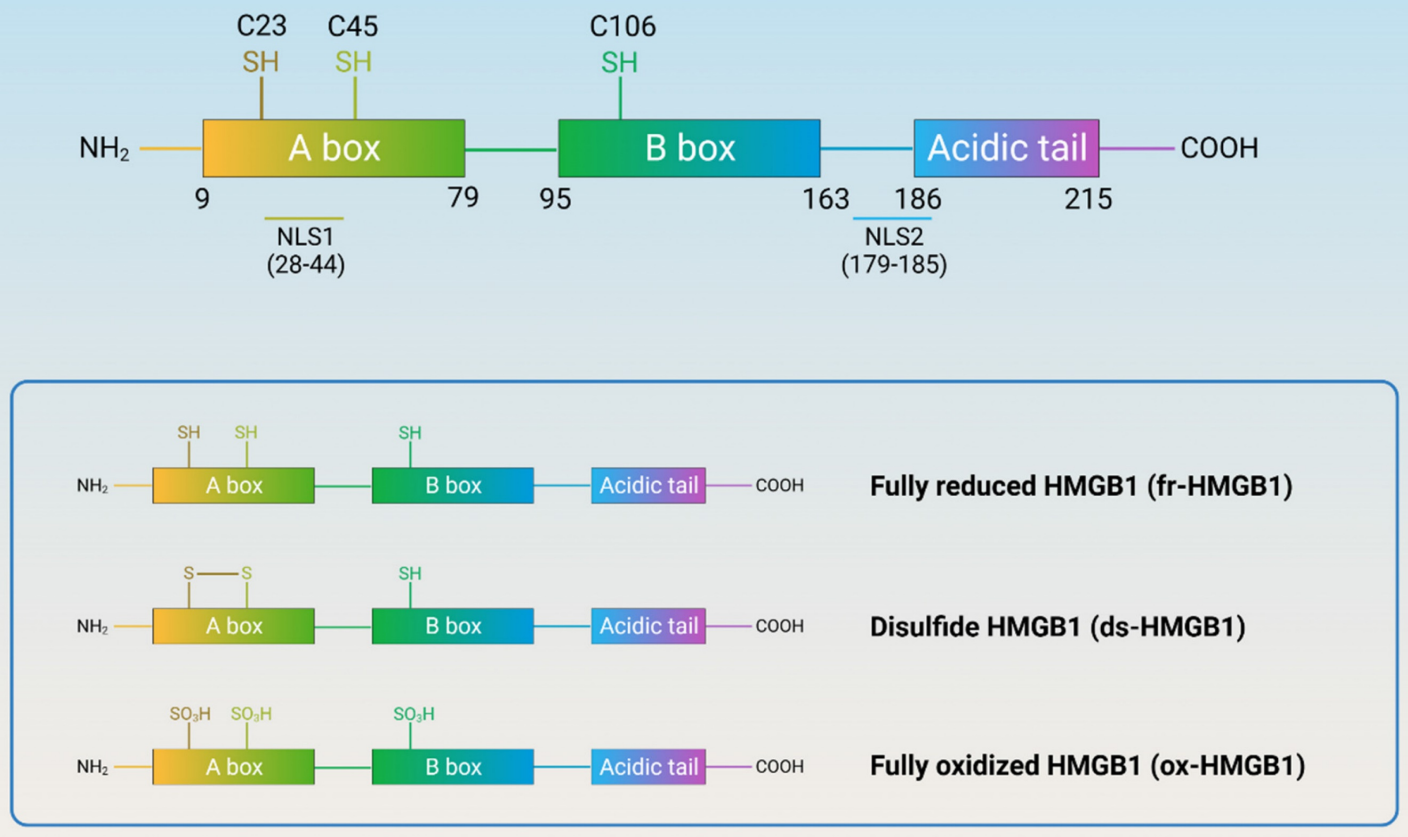
** Structure and redox reaction of HMGB1.** (HMGB1 is composed of A-box, B-box, C-terminal acidic tail, and a short but functionally significant N-terminal region, with nuclear localization signals and three redox-sensitive cysteine residues. HMGB1 can be classified into three subtypes: fully reduced HMGB1, disulfide HMGB1, and fully oxidized HMGB1.)

Human HMGB1 consists of 215 amino acid residues that form two homologous DNA-binding domains (A-box and B-box), a negatively charged C-terminal acidic tail, and a short but functionally significant N-terminal region (Figure 2). HMGB1 contains three redox-sensitive cysteine residues (C23, C45, and C106). C23 and C45 can form intramolecular disulfide bonds, while C106 is unpaired. Based on the redox status of the three cysteine residues, HMGB1 can be classified into three subtypes: fully reduced HMGB1 (fr-HMGB1, with three conserved cysteine residues containing thiol groups), disulfide HMGB1 (ds-HMGB1, partially oxidized), and fully oxidized HMGB1 (ox-HMGB1, sulfonyl HMGB1). fr-HMGB1 can bind to other chemokines to promote immune cell migration and tissue regeneration. ds-HMGB1 can activate immune cells to produce cytokines/chemokines and exhibit a higher affinity for the nuclear export of CRM1^13^. However, ox-HMGB1 exhibited no chemokine or cytokine activity. Importantly, the exchange between fr-HMGB1 and ds-HMGB1 is reversible, while that with ox-HMGB1 is irreversible^14^.

### 2.2. The distribution and function of HMGB1

**Figure 3:**
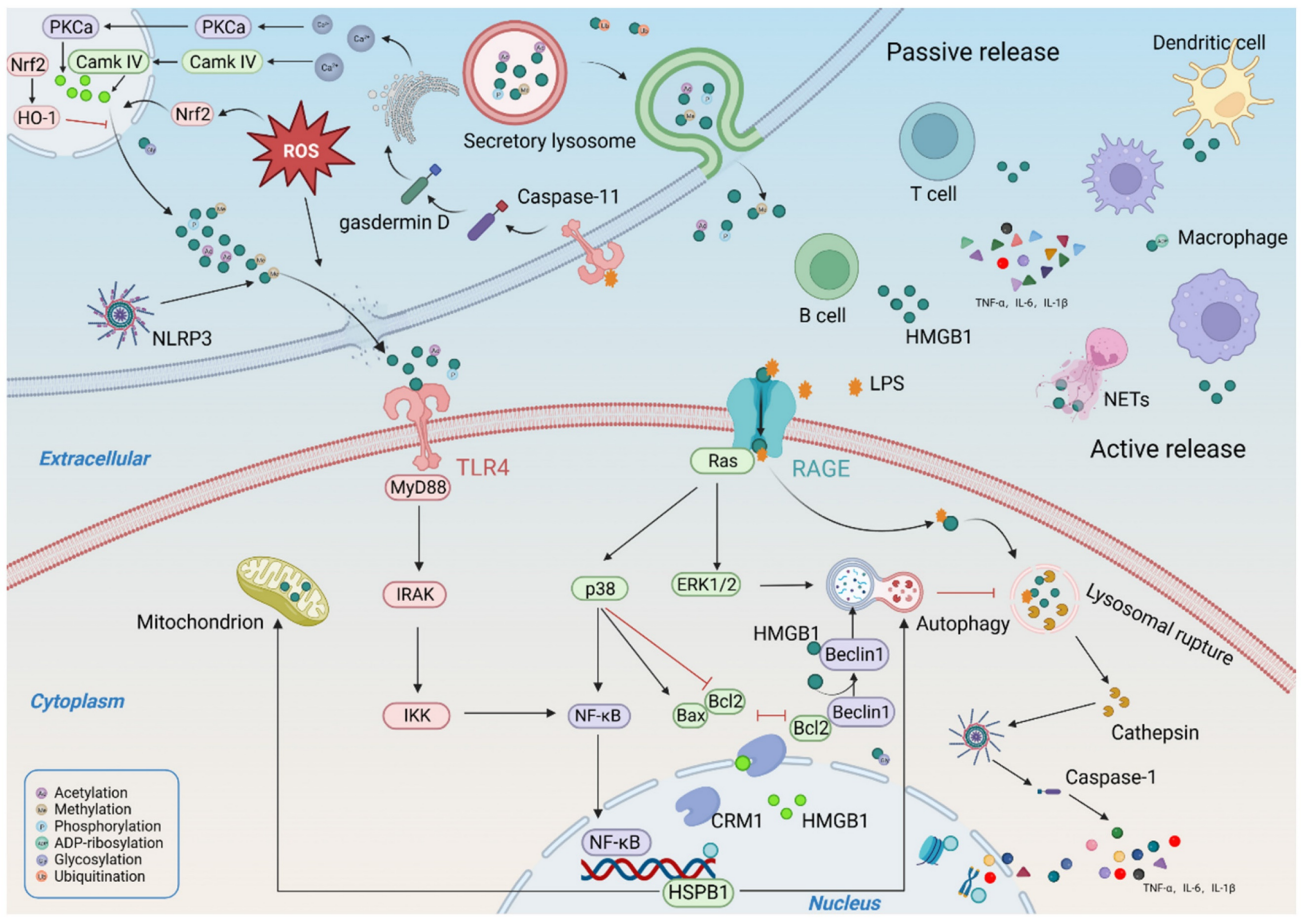
** The distribution and function of HMGB1.** (HMGB1 can cross organelles from the nucleus at higher concentrations into the cytoplasm in response to stress injury. The function of HMGB1 is related to its subcellular structure. In the nucleus, HMGB1 plays an important role in DNA replication and repair, chromatin remodeling, nucleosome assembly, and telomere maintenance; In the cytoplasm, HMGB1 is primarily involved in regulating autophagy, mitochondrial function, and apoptosis; Extracellular HMGB1 primarily serves as a DAMP and participates in many immune responses, can also promote cell migration and proliferation.)

HMGB1 is highly expressed in various kidney cells, and its role in CKD pathogenesis depends on its subcellular localization (Figure 3). In the nucleus, HMGB1 promotes the repair of damaged DNA and the maintenance of nucleosome homeostasis and telomere homeostasis. In particular, the retention of HMGB1 in the nucleus improves the differentiation of peripheral B cells and the phagocytic capacity and chemotactic response of macrophages^15^, ^16^. In the cytoplasm, HMGB1 is primarily involved in regulating autophagy, mitochondrial function, and apoptosis. Extracellular HMGB1 primarily serves as a DAMP and participates in many immune responses by promoting immune cell maturation, activation, and cytokine production^17^. More importantly, extracellular HMGB1 is associated with cell death. extracellular HMGB1 can be internalized and targeted to lysosomes, inducing lysosomal membrane permeabilization (LMP) and accelerating subsequent cell death^18^, ^19^.

### 2.3. The modification and regulation of HMGB1

The localization and activity of HMGB1 are affected by PTMs^20^, including acetylation, methylation, phosphorylation, poly-ADP-ribosylation, and glycosylation. Acetylation enhances the ability of HMGB1 to bend DNA and prevents HMGB1 from re-entering the nucleus^21^, ^22^. Resveratrol (a natural SIRT1 agonist) pretreatment promoted the nuclear retention of HMGB1 by reducing HMGB1 acetylation, thereby improving renal inflammation and tubular injury^23^. Methylation changes the conformation of HMGB1 and weakens its DNA-binding activity, allowing its massive passive diffusion into the cytoplasm and subsequent secretion extracellularly^24^. Phosphorylation also limits the nuclear localization of HMGB1 by modifying its two NLS 25. Poly (ADP)-ribose polymerase (PARP) also promotes the nuclear release of HMGB1 into the extracellular environment^26^. Poly-(ADP)-ribosylated HMGB1 not only downregulates gene transcription^27^, ^28^ but also inhibits efferocytosis in macrophages to a significant extent, thereby promoting inflammation^29^. In turn, the deletion of HMGB1 leads to excessive PARP-1 activation, which exacerbates mitochondrial damage and cell death^30^. In addition, PARP-1 also induced the release of HMGB1 from proximal tubular cells^31^. Glycosylation plays a crucial role in HMGB1secretion. N-glycosylation weakens the binding of HMGB1 to DNA and enhances its binding to the nuclear export protein CRM1, a prerequisite for HMGB1 cytoplasmic transport and extracellular secretion^32^. In addition, N-glycosylation of HMGB1 leads to reduced binding to glycyrrhizin, an HMGB1 inhibitor^33^. Recent studies have demonstrated that O-glcnacylation can also modify HMGB1, resulting in its reduced ability to repair DNA^34^. Recently, S-nitrosylation has been shown to promote HMGB1 secretion and proinflammatory effects^35^. Ubiquitination modification helps promote HMGB1 degradation and improves disease progression^36^. Ubiquitin-specific protease-12 deubiquitinates and stabilizes HMGB1 to promote autophagy by interacting with HMGB1^37^. However, another study showed that compared with the ubiquitination pathway, the autophagy-lysosome pathway plays a major role in HMGB1 degradation, and the activation of autophagy and an increase in CTSB promote HMGB1 degradation and nuclear translocation^38^.

### 2.4. Secretion and release of HMGB1

Under the action of various stressors (such as hypoxia, cytokines, chemokines, and uremic toxins) in CKD, HMGB1 is secreted externally through an unconventional protein secretion pathway in an active or passive manner, rather than through the conventional endoplasmic reticulum-Golgi pathway^6^. At present, two main secretion pathways of HMGB1 have been proposed. One is the direct, pore-mediated secretion of HMGB1 by pyroptosis or activated target cells^39^; the other is secretory autophagy, which packages HMGB1 into intracellular vesicles (such as lysosomes or autophagosomes) and releases HMGB1 through exocytosis^40^. However, these two pathways are difficult to distinguish because they occur simultaneously in most pyroptotic cells^39^. The regulatory mechanism underlying HMGB1 secretion is complex and involves several pathways. Oxidative stress is known to be an important factor in regulating HMGB1 secretion^41^. HMGB1 is secreted through a ROS-dependent mechanism under hypoxia, and targeted inhibition of ROS production significantly reduces HMGB1 secretion. Nuclear factor erythroid 2-related factor 2 (Nrf2) is a key transcription factor regulated by oxidative stress. Nrf2 knockdown abolishes the regulatory effect of antioxidants on HMGB1^42^. Notably, the regulation of HMGB1 secretion by oxidative stress is driven by calcium signaling^43^. The inhibition of calcium/calmodulin-dependent kinase resulted in a significant reduction in HMGB1 secretion. Calcium overload promotes the release of phosphorylated HMGB1^44^. Moreover, HMGB1 release mediates calcium influx by promoting calcium channel activation 45. In addition, the cytoplasmic translocation and secretion of HMGB1 are also tightly regulated by the nuclear export protein CRM1. The inhibition of CRM1 expression significantly reduced circulating HMGB1 levels^46^. In addition, several cathepsin family members also promote HMGB1 secretion. Under stress, the permeability of the lysosomal membrane is altered, leading to the release of cathepsins and other hydrolases in the cytoplasm and their subsequent translocation to the nucleus, inducing the formation of the NLRP3 inflammasome complex, ultimately leading to increased HMGB1 secretion^47^, ^48^. The released HMGB1 targets LPS internalization into lysosomes through RAGE and mediates lysosomal leakage, which activates caspase 11 and promotes pyroptosis^18^. Targeted inhibition of HMGB1 binding to LPS improves lysosomal rupture and attenuates caspase 11-mediated sepsis-related lethality^49^. A recent study showed that TLR4 also increases the expression of caspase 11 through LPS uptake and that activated caspase 11 promotes the cleavage of gasdermin D, resulting in increased calcium release from the endoplasmic reticulum, which in turn promotes HMGB1 secretion^50^.

## 3. HMGB1 and renal homeostasis

**Figure 4:**
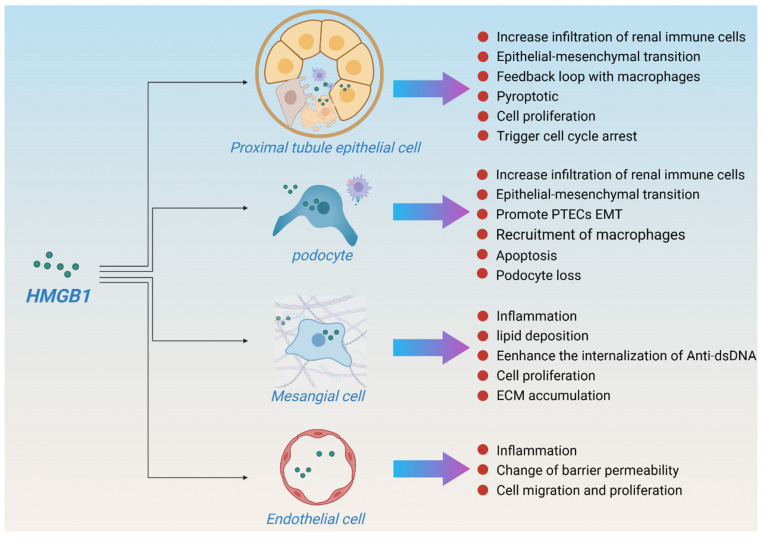
** HMGB1 and renal homeostasis.** (HMGB1 is expressed in a variety of kidney cell types, especially in proximal tubule epithelial cell and podocyte. In case of injury, renal tubular epithelial cells and podocytes are the main sources of HMGB1, and mesangial and endothelial cells also express HMGB1. HMGB1 mediates kidney damage and repair through multiple pathways to maintain renal homeostasis.)

HMGB1 is expressed in a variety of kidney cell types, including glomerular epithelial cells (podocytes), endothelial cells, tubular cells, inflammatory mononuclear phagocytes, and lymphocytes. In case of injury, renal tubular epithelial cells and podocytes are the main sources of HMGB1, and mesangial and endothelial cells also express HMGB1^51^, which in turn promotes apoptosis and renal inflammation. Although macrophage-derived HMGB1 plays an important role in many diseases^52^, ^53^, studies have shown that macrophage-derived HMGB1 does not aggravate renal fibrosis after UUO^54^. In contrast, the deletion of bone marrow-derived RAGE contributed to the improvement of renal function in a DKD mouse model^55^, indicating that macrophages may only be effectors of HMGB1 rather than the main secretory source during kidney injury, especially in CKD. In this section, we summarize the important regulatory effects of HMGB1 on various intrinsic kidney cell types to maintain kidney homeostasis. The effects of HMGB1 on intrinsic renal cells in various kidney disease models are summarized in Table 1 and Figure 4.

### 3.1 HMGB1 and proximal tubule epithelial cell

Proximal tubular epithelial cells (PTECs) are the major epithelial cell type in the cortex. The effects of HMGB1 on PTECs function have been extensively studied. HMGB1-mediated tubular injury and renal fibrosis are seemingly hallmarks of chronic processes^10^. In the early stages of PTEC injury, HMGB1 secretion promotes rapid γδ T-cell infiltration and mediates an early immune response to renal injury^56^. In the late stage of kidney injury, PTECs reduce HMGB1 secretion through locally produced propertin (a positive regulator of the alternative complement pathway), thereby reducing macrophage infiltration and enhancing the phagocytic capacity of PTECs, which in turn curtails apoptosis and kidney inflammation^57^. In addition, free light chains (FLCs) promote the secretion of HMGB1 by PTECs and the expression of TLR2, TLR4, and TLR6, resulting in an overload of the endocytic pathway of FLCs, which triggers inflammation and cell damage^58^. HMGB1 also promotes transforming growth factor β (TGF-β) and connective tissue growth factor (CTGF) expression and induces epithelial-mesenchymal transition (EMT) of PTECs, ultimately accelerating renal fibrosis^59^-^61^.

Notably, HMGB1 is involved in renal tubular injury caused by various nephrotoxic drugs. For instance, imidacloprid stress induces Nrf2 inactivation and mediates HMGB1/RAGE/TLR4 signaling activation, thereby triggering iron death and leading to the initial wave of death that fuels pyroptosis and exacerbates renal dysfunction^62^. Similarly, cyclosporine, aristolochic acid I, and calcineurin inhibitors also promote HMGB1 secretion by tubular cells, aggravating tubular injury and renal fibrosis^63^-^65^, suggesting that HMGB1 might serve as an early indicator and marker of progressive nephrotoxicity.

Although neutralization of extracellular HMGB1 is beneficial, intracellular HMGB1 seems to play an additional role in renal tubular injury^51^. The induction of increased HMGB1 levels in the plasma and urine by remote ischemic preconditioning, but not by increased infiltration of renal immune cells, reportedly reduced the risk of renal injury^66^. Indeed, recent studies have also found that HMGB1 plays a dual role in renal tubules. It has been found that HMGB1 binds to TLR4 on PTECs to trigger transient protective G1 cell cycle arrest, providing renal protection^67^, while TLR4 activation on non-renal cells has been shown to contribute to renal injury^66^.

### 3.2 HMGB1 and podocyte

Podocytes are terminally differentiated glomerular epithelial cells that play a key role in maintaining the glomerular filtration barrier^68^. Podocytes are non-professional antigen-presenting cells that are both the target of inflammatory injury and active participants^69^. Damaged podocytes are one of the primary sources of renal HMGB1 secretion. Under injury conditions, podocytes promote renal injury by secreting HMGB1 to promote the EMT of PTECs, mitochondrial damage, and apoptosis^70^. Targeting HMGB1 inhibition or depletion ameliorates podocyte injury and EMT by regulating autophagy homeostasis^71^. CLEC14a is a single-pass transmembrane glycoprotein that exerts a protective effect on podocytes. CLEC14a ameliorates podocyte injury by improving NF-κB signaling and early growth response protein 1 signaling via directly binding to HMGB1 and inhibiting its release^72^. In addition, deleting bone marrow-derived RAGE improved podocyte loss following streptozocin (STZ) induction^55^.

### 3.3 HMGB1 and mesangial cell

Mesangial cells (MCs) play an important role in maintaining the structural integrity of the glomerular microvascular bed and mesangial matrix homeostasis^73^. HMGB1 is an important mediator of MC activation. HMGB1 mediates lipid deposition in MCs by promoting the transcription and expression of sterol regulatory element-binding protein-1 and fatty acid synthase 74. In lupus nephritis (LN), HMGB1 also enhances the internalization of anti‐double‐stranded DNA (dsDNA) IgG in MCs by binding to dsDNA IgG and activates the MyD88/NF-κB pathway^75^, ^76^, which exhibits a synergistic proinflammatory effect that mediates the activation of MCs^77^, leading to renal tubular cell death and increased cytokine release^78^, thereby aggravating proteinuria, glomerulosclerosis, and renal fibrosis in LN^76^, ^79^. The depletion of HMGB1 in MCs inhibits iron death and improves MC proliferation by regulating Nrf2 signaling^80^.

### 3.4 HMGB1 and endothelial cell

The population of ECs in the kidney is remarkably diverse, and approximately 24 morphologically and functionally heterogeneous EC types have been identified^81^. ECs are extremely sensitive to stress, and disruption of endothelial function is considered an early event in kidney injury^82^. At the early stage of kidney injury, HMGB1 released first exacerbates kidney injury by interacting with TLR4 in renal ECs (much earlier than in renal tubular epithelial cells), leading to EC activation and upregulating the expression of adhesion molecules^83^. HMGB1 is an important regulator of ECs. On the one hand, HMGB1 induces a proinflammatory response in ECs, leading to early changes in barrier permeability in ECs. On the other hand, the internalization of HMGB1 into ECs promotes the expression of vascular endothelial growth factor, which in turn promotes EC migration and proliferation^84^, ^85^. HMGB1 is highly expressed in the glomerular ECs of patients with LN. HMGB1 promotes the permeability of ECs and the shedding of the glycocalyx in the glomerulus and disrupts intercellular tight junctions and cytoskeleton arrangement, thus aggravating LN-related proteinuria^86^. Similarly, HMGB1 mediates myeloperoxidase (MPO)-antineutrophil cytoplasmic antibody (ANCA)-induced EC activation and glomerular damage by triggering moesin phosphorylation and secretion and promoting cross-reactivity between moesin and the anti-MPO antibody^87^.

## 4. Pathogenic roles of HMGB1 in CKD

**Figure 5:**
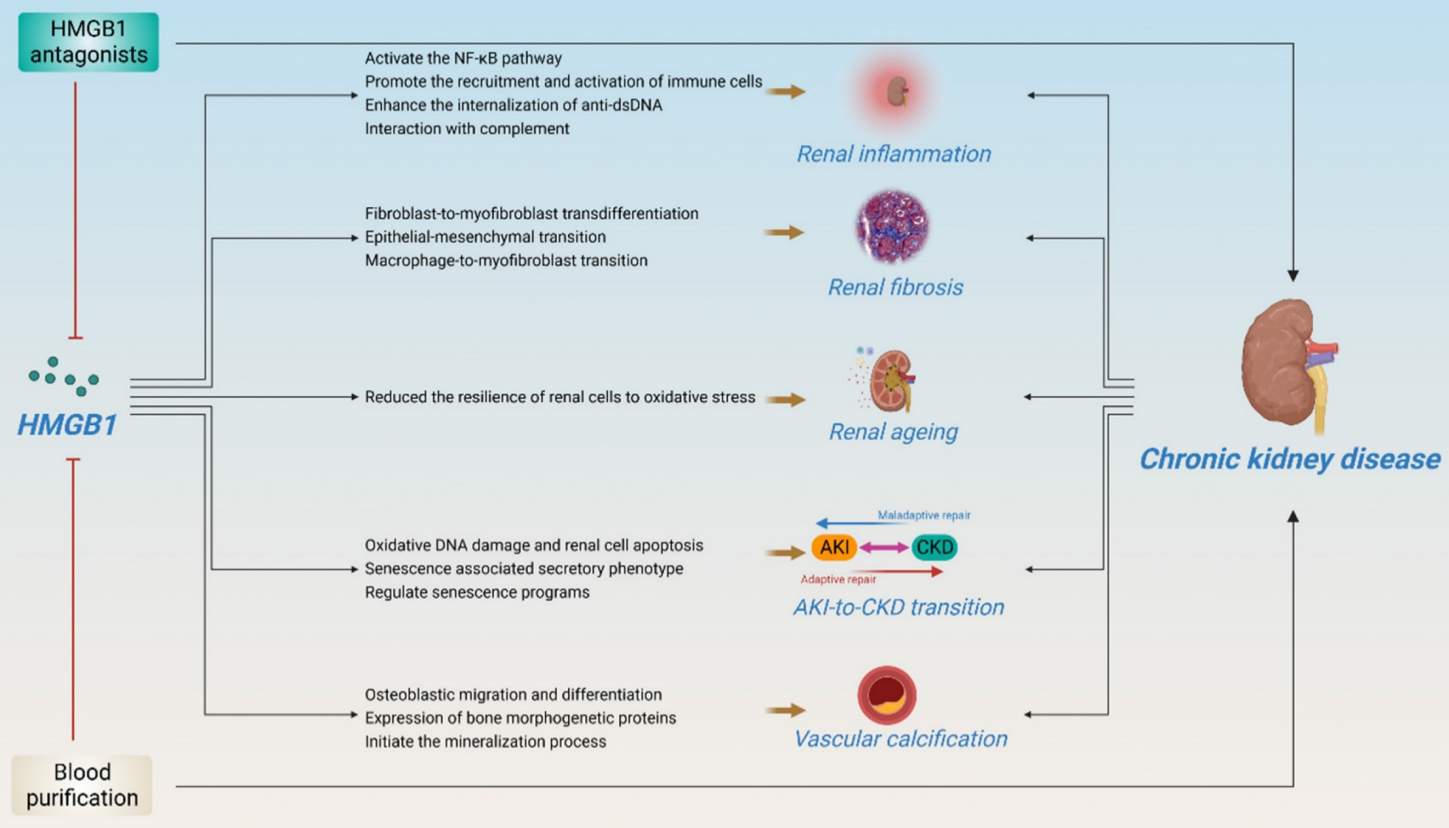
** Pathogenic roles of HMGB1 in CKD.** (HMGB1 plays an important role in kidney disease, especially in CKD, including kidney inflammation, fibrosis, ageing, AKI-to-CKD transition, vascular calcification, and renal replacement therapy. Several HMGB1 inhibitors and hemodialysis have shown potential therapeutic effects in improving CKD.)

HMGB1 is involved in kidney disease progression. Although the important role of HMGB1 in kidney disease has been elegantly described in several reviews^88^, ^89^, recent research advances suggest that HMGB1 plays an important role in kidney disease, especially in CKD, including kidney inflammation, fibrosis, ageing, AKI-to-CKD transition, vascular calcification, and renal replacement therapy, anticipating that strategies to block the interaction between HMGB1 and its receptor may be effective in preventing the development of CKD (Figure 5).

### 4.1 The clinical value of HMGB1 in CKD

HMGB1 has been confirmed to be associated with the occurrence, progression, and prognosis of CKD in multiple clinical studies (Table 2). A study including 177 CKD patients found that HMGB1 was significantly elevated in patients with CKD and correlated with estimated glomerular filtration rate (eGFR) and markers of inflammation and malnutrition^90^, ^91^. An observational study including 20 patients with non-diabetic nephropathy found that serum HMGB1 was significantly elevated in CKD and independently correlated with the accumulation of asymmetric dimethylarginine, indicating that HMGB1 is actively involved in CKD progression and might lead to the development and progression of cardiovascular diseases (CVDs)^92^. In terms of pathological typing, a study including 258 patients with chronic glomerulonephritis (GN) found that HMGB1 tended to be significantly elevated in the serum of patients with ANCA-GN, Henoch-Schonlein purpura nephritis, and IgA nephropathy with glomerular crescents^93^.

In addition, HMGB1 shows good discriminative ability for LN. Serum HMGB1 levels are significantly elevated in patients with LN and correlate with SLE activity^94^, ^95^. Serum HMGB1 levels also positively correlate with proteinuria in patients with LN 94, 95. However, in another study involving 69 patients with SLE, no significant correlation was found between serum HMGB1 and proteinuria 96. In the urine of patients with LN, HMGB1 was also significantly elevated and correlated with the LN class, with higher levels of urinary HMGB1 in patients with LN class V^97^. Another study found that microparticle (MPs)-HMGB1 was elevated in the circulation and urine of patients with LN, and MP-HMGB1 in urine showed good discriminative ability for the presence of LN and disease activity^98^. Notably, at the end of follow-up, immunosuppressive treatment only reduced HMGB1 expression in the serum and renal tissues of class IV LN patients, whereas HMGB1 levels in other patients with LN did not change significantly before and after treatment, possibly due to persistent chronic inflammation^99^.

The correlation between HMGB1 and renal injury in patients with ANCA-associated vasculitis (AAV) is particularly strong. A study that collected plasma samples from 74 patients with active AAV and 65 patients with remission AAV found that circulating HMGB1 levels were associated with renal involvement and that plasma HMGB1 levels significantly correlated with initial serum creatinine and eGFR^100^. Similarly, in a study that included 30 patients with AAV, HMGB1 was significantly elevated in AAV patients with renal involvement and continued to increase with disease activity^101^. In a study including 51 patients with AAV, serum HMGB1 was found to correlate significantly with disease activity and renal involvement and positively correlated with serum creatinine and 24-hour urinary protein levels in patients with AAV^102^. Similarly, urine HMGB1 levels also are associated with renal involvement in patients with AAV 103.

### 4.2 Renal inflammation and fibrosis

Patients with CKD experience persistent inflammation in the early stages, which determines the progression of most kidney diseases. Cells exposed to the kidney disease environment undergo phenotypic changes and overproduce proinflammatory cytokines, which in turn contribute to the recruitment of cells involved in innate and adaptive immune responses, further amplifying inflammation and damage to the kidney^104^. HMGB1 is an emerging mediator of renal inflammation. Mechanistically, On the one hand, HMGB1 activates the NF-κB pathway by interacting with RAGE and TLR4 in kidney cells^105^, ^106^; On the other hand, by promoting the recruitment and activation of immune cells, including macrophages^107^, dendritic cells and B cells^108^. In LN, the released HMGB1 also contributes to the endocytosis of extracellular accumulated DNA and the activation of cyclic GMP-AMP synthase signaling pathway, and the subsequent secretion of IFN-I, leading to the expansion of downstream inflammation^109^. In addition, HMGB1 also amplifies renal inflammation through the interaction with complement. In ANCA, HMGB1 promoted C5a-mediated translocation of ANCA antigens and neutrophil activation, thereby aggravating renal involvement^110^. Importantly, HMGB1 mediated inflammation is an important factor driving renal fibrosis^111^. The severity of renal fibrosis is positively correlated with the activation of HMGB1/TLR2/TLR4 signaling^112^. Specifically, HMGB1 promotes the expression of TGF-β and CTGF by activating multiple inflammatory pathways^113^, ^114^, which in turn promotes fibroblast-to-myofibroblast transdifferentiation and EMT^115^, ^116^, accelerating renal fibrosis. In addition, HMGB1 promote the recruitment and activation of macrophages in the early stages of UUO and induce macrophage-to-myofibroblast transition, thereby promoting renal fibrosis^117^. Interestingly, surfactant protein A, a novel protein factor, can block TGF-β1 expression and renal fibroblast transdifferentiation by binding HMGB1, thereby improving renal fibrosis^115^. Therefore, targeted inhibition of HMGB1 may be a good strategy for improving renal fibrosis.

### 4.3 AKI-to-CKD transition

AKI leads to a significantly higher risk of CKD and ESRD, as well as higher mortality^118^. Therefore, preventing the transition from AKI to CKD is essential. Maladaptive repair and increased irreversible renal fibrosis after AKI are the main causes of CKD^119^, including tubular epithelial cell injury, endothelial dysfunction, microvascular rarefaction, and inflammatory progression. Recent evidence suggests the significant role of HMGB1 in the AKI-to-CKD transition. HMGB1 is a driver of necroinflammation in AKI. Although neutralizing extracellular HMGB1 is beneficial for renal protection, HMGB1 knockdown provides additional renal protection, indicating that intracellular HMGB1 has an extracellular-independent effect^51^. Further studies have shown that intracellular HMGB1 reduces the resistance of renal tubular cells to oxidative stress^51^. The inhibition or deletion of intracellular HMGB1 promotes the proliferation and regeneration of injured renal tubular epithelial cells and reduces renal interstitial matrix deposition and neutrophil gelatinase-associated lipocalin expression, thereby improving the AKI-to-CKD transition^51^. Therefore, intracellular HMGB1 may be a potential target for enhancing kidney regeneration and improving the long-term prognosis of AKI.

### 4.4 Renal aging

Kidney aging increases vulnerability to disease. At the cellular level, senescence causes cells to be in a permanent and irreversible cell cycle arrest and secrete a series of proinflammatory cytokines and growth factors, known as the SASP^120^. In a D-galactose-induced age-related renal injury model, HMGB1 expression was significantly increased, accompanied by enhanced oxidative DNA damage and renal cell apoptosis^121^. HMGB1, through the NF-κB signaling pathway activation, promotes the crosstalk between the high expression of inflammatory factors and premature senescence of renal cells to play its role in renal injury^122^, ^123^. A recent study reported that nuclear HMGB1 directly binds to topologically associated domains or RNA to regulate proliferation or senescence 124. In addition, HMGB1 consolidates DNA durability by increasing gaps in DNA, leading to DNA protection and improved cellular senescence^125^.

### 4.5 Vascular calcification in CKD

Vascular calcification (VC) is an important factor contributing to CVDs-related morbidity and mortality in CKD^126^. In CKD, the accumulation of uremic toxins, oxidative stress, and chronic inflammation induces an imbalance in calcium and phosphate homeostasis and the transformation of vascular smooth muscle cells into chondrocytes or osteoblast-like cells, ultimately leading to VC^127^. HMGB1 might plausibly play a crucial role in VC in CKD. On the one hand, HMGB1 promotes osteoblastic migration and differentiation by activating RAGE/TRL4 signaling pathway^128^, ^129^. On the other hand, HMGB1 induces calcium deposition by regulating the expression of bone morphogenetic proteins^130^. In addition, HMGB1 reportedly initiates the mineralization process by promoting the secretion of extracellular matrix vesicles by macrophages, leading to shifted pathological mineralization^131^. Interestingly, the expression of osteopontin proteins and mineral particles promotes the cytoplasmic translocation and secretion of HMGB1^132^, ^133^. In a 5/6 nephrectomy-induced CKD model, a high-phosphate diet triggered inflammatory aortic calcification by promoting the nuclear-cytoplasmic translocation of HMGB1 in aortic tissue and inducing the expression of Runx2, osteopontin, and Msx2^134^. In addition, VC is also associated with the activation of Wnt/β-catenin pathway. HMGB1 promotes VC by activating the β-catenin pathway, upregulating Runx2, and downregulating Klotho in CKD^91^. Bone marrow mesenchymal stem cell-derived exosomes improve aortic calcification by promoting SIRT6 expression and reducing HMGB1 cytoplasmic translocation via deacetylation^134^. In addition, lethal giant larvae 1, a key regulator of cell polarity, can also inhibits calcification by binding to HMGB1 and promoting its degradation through the lysosomal pathway^135^.

### 4.6 Renal replacement therapy

Renal replacement therapy or kidney transplantation is the cornerstone of patient with ESRD treatment. HMGB1 is a late inflammatory mediator in CKD. HMGB1 levels are significantly elevated in patients undergoing continuous ambulatory peritoneal dialysis (CAPD) and are associated with inflammation and malnutrition^136^. Released HMGB1 mediates peritoneal fibrosis during peritoneal dialysis (PD) treatment by promoting MCP-1 and IL-8 production^137^. Serum HMGB1 levels were significantly higher in patients undergoing hemodialysis than in patients with PD. Of note, HMGB1 levels decrease significantly with dialysis treatment^138^. Interestingly, patients with higher HMGB1 levels face more complications than those with lower HMGB1 levels despite no difference in terms of survival^138^. HMGB1 can be cleared by hemofiltration and hemodialysis using super-high-flux or high-cutoff membranes^139^. Therefore, the targeted clearance of HMGB1 by *in vitro* blood purification might effectively improve the clinical outcomes of critically ill patients, including ESRD^140^.

## 5. HMGB1 antagonists of potential clinical interest in CKD

At present, several strategies have been shown to successfully inhibit HMGB1-dependent diseases, including inhibiting HMGB1 expression and release, as well as blocking HMGB1-related signaling (HMGB1/TLR4 or HMGB1/RAGE pathway) (Table 3)^7^. Targeted HMGB1 therapy has been widely studied and applied to many diseases. In CKD, Ethyl pyruvate (EP), a well-established and potent HMGB1 inhibitor, selectively inhibits HMGB1 translocation from the nucleus, which inhibits its function in the cytosol and the active secretion of HMGB1 upon cell activation^141^. EP ameliorated albuminuria and glomerular injury in an STZ-induced DKD rat model by inhibiting HMGB1^142^ and alleviated CaCl_2_-induced renal tubular cell injury by downregulating the expression of inflammatory and autophagic proteins^143^. Glycyrrhizic acid (Gly) was the first natural HMGB1 inhibitor to be discovered. Gly induces conformational changes that interfere with the DNA-binding ability of HMGB1 in the nucleus, HMGB1 phosphorylation in the cytosol, and the binding ability of HMGB1 receptors in the extracellular space^144^. Gly ameliorates proteinuria, pathological renal injury, and disease progression in DKD rats by improving renal inflammation and ROS production by inhibiting HMGB1^145^-^147^. Gly can also prevent tacrolimus-induced renal injury by improving lysosomal function and regulating autophagy^148^. Many other natural products, such as *Korea red ginseng*^121^, Bupleurum polysaccharides^149^, Dioscin^150^, ^151^, *Plantago asiatica L*^152^, Isomangiferin^153^, Troxerutin^154^, and Ellagic acid^155^, have been found to have similar therapeutic effects on CKD.

Anti-HMGB1 antibodies have also been shown to fully inhibit the increase in complement deposition and albuminuria in MRL/lpr lupus-prone mice by inhibiting neutrophil recruitment and NETs^156^, ^157^. Anti-HMGB1 antibody administration inhibits NF-κB expression by blocking the activation of the TLR4 pathway, thereby improving tubulointerstitial fibrosis, improving serum creatinine and 24-hour albuminuria, reducing creatinine clearance associated with nephrotoxicity, and preventing calcineurin inhibitor-induced nephrotoxicity, which is beneficial for improving the allograft survival rate of renal transplant recipients^158^. The supra-physiological production of endogenous secretory RAGE or administration of the HMGB1 A-box also improved albuminuria, glomerular injury, interstitial fibrosis, and renal inflammation in DKD mice^159^.

In addition, some drugs that have proven effective in treating CKD were found to be associated with HMGB1 inhibition. For instance, the renoprotective effect of empagliflozin alleviated renal inflammation and apoptosis and was associated with reduced levels of HMGB1, RAGE, and TLR4^160^. The renoprotective effect of dapagliflozin is related to the blocking of the renal HMGB1 feedback loop^161^. Dapagliflozin alleviates renal tubular injury, improves inflammation and oxidative stress^162^, and reverses podocyte loss and fibrosis by restoring renal autophagy by inhibiting HMGB1 in DKD^163^. Simvastatin ameliorated pathological renal injury by inhibiting HMGB1 expression in the kidneys of LN mice^164^.

Interestingly, with the development of computational tools, new HMGB1 inhibitors (such as nano selenium and sildenafil), have shown satisfactory effects in improving renal function and pathological damage^165^. Based on the above evidence, HMGB1 may be an attractive target for the treatment of CKD. Nevertheless, more efficient and safer HMGB1 inhibition strategies are urgently required to improve the therapeutic effects on CKD.

## 6. Conclusions and perspectives

HMGB1 plays multiple roles in the occurrence and progression of CKD depending on its localization, context, post-translational modification, and receptor binding. HMGB1 is expressed and secreted by stressed intrinsic renal cells and mediates renal fibrosis, aging, AKI-to-CKD transition, and cardiovascular complications by amplifying inflammation through the regulation of autophagy- and cell death-related pathways, ultimately affecting renal outcomes. In addition, as a biomarker, HMGB1 levels also significantly correlate with the progression and prognosis of CKD. Pharmacological inhibition and deletion of HMGB1 significantly improve various kidney disease phenotypes. Therefore, targeting HMGB1 is an attractive therapeutic strategy for CKD treatment. However, applying HMGB1 as a therapeutic target in CKD remains challenging. The first issue is the accurate measurement of HMGB1 levels. Studies have confirmed that HMGB1 is produced in serum during blood clots 166. Therefore, whether plasma, serum, or urine is the best sample to predict and evaluate CKD warrants further study. In addition, studies have found that HMGB1 binds to several proteins, including IgG1, in the serum to form a complex that interferes with the enzyme linked immunosorbent assay system detection^167^. Therefore, it is important to clarify whether western blot, ELISA, liquid chromatography and tandem mass spectrometry, and other alternative methods can accurately detect HMGB1. Another critical issue that needs to be addressed is the heterogeneity in HMGB1 expression. Studies have shown sex differences in HMGB1 expression in kidney injury and that HMGB1 increases more in male rats upon kidney injury^168^. In addition, HMGB1 expression is tissue-specific^169^. Therefore, clarifying the specific factors that affect the differences in HMGB1 expression might help the development of targeted treatments for HMGB1. Finally, the decrease in renal clearance does not fully explain the increase in circulating HMGB1. Although splenectomy transiently reduced circulating HMGB1 levels and improved CKD. However, the source of HMGB1 in CKD remains to be elucidated^170^. Furthermore, at the cellular level, the dual localization of HMGB1 appears to be functionally complementary. How damaged renal intrinsic cells balance the nuclear and extracellular functions of HMGB1 remains unclear, especially, what role does intracellular HMGB1 play, and whether extracellular HMGB1 is the cause or result of kidney injury, which is the premise for identifying highly effective HMGB1 inhibitors for CKD.

## Funding

This study was supported by the National Nature Science Foundation of China (82374419, 82074393, 82305210).

## Figures and Tables

**Table 1:** HMGB1 effects in renal resident cells.

Cell types	Types of study	Experimental models	Pathway	HMGB1-mediated effects	References
Proximal tubule epithelial cell	*In vitro, In vivo*	UUO	TNFα/Casp3/GSDME/HMGB1	Promote inflammation, PTEC damage and fibrosis	10
	*In vitro, In vivo*	CsA-induced renal injury	HMGB1/TLR4	Promote inflammation and fibrosis	63
	*In vivo*	HG-induced HK-2	HMGB1/TLR4/Syk	Promote NF-κB activation and TGF-β1 production	171
	*In vitro, In vivo*	Ang II-induced renal injury	NLRP3/HMGB1	Promote EMT and fibrosis	172
	*In vitro, In vivo*	FLCs-induced renal injury	STAT1/HMGB1/TLR	Promote inflammation and PTEC damage	58
	*In vivo*	AGE-induced HK-2	HMGB1/RAGE	Promote the expression of CTGF and TGF-β	59
	*In vivo*	HMGB1-induced HK-2	HMGB1/RAGE	Promote EMT	60
	*In vitro, In vivo*	UUO	C3 / HMGB1 / TGF-β1	Promote EMT and fibrosis	61
	*In vitro, In vivo*	STZ-induced DKD	HMGB1/TLR2/4/NF-κB	Promote inflammation	161
	*In vitro, In vivo*	HFD-fed OLETF rats	NLRP3/HMGB1	Promote inflammation and PTEC damage	173
	*In vitro, In vivo*	IMI-induced renal injury	HMGB1-RAGE/TLR4-NF-κB	Promote PTEC Ferroptosis and pyroptosis	62
	*In vivo*	AAs-induced HK-2	ROS/HMGB1/mt DNA/ TLRs	EMT and mitochondrial dysfunction	64
	*In vitro, In vivo*	CNIs-induced renal injury	/	Promote PTEC mitochondrial dysfunction and bioenergetic reprograming	65
	*In vivo*	CaCl_2_-induced HK-2	HMGB1/TLR4/NF-κB	Promote inflammation and autophagy	143
	*In vitro, In vivo*	UUO	/	Promote fibrosis	115
Podocyte	*In vitro, In vivo*	db/db mice	/	Promote podocyte apoptosis and EMT	71.
	*In vitro, In vivo*	ADR-induced renal injury	/	Promote podocyte injury and proteinuria	72
Mesangial cell	*In vitro, In vivo*	MRL/lpr mice	TLR2/MyD88/NF-κB	Promote glomerular mesangial matrix deposition	76
	*In vivo*	HG-induced SV40 MES 13	HMGB1/NF-κB	Promote inflammation	174
	*In vivo*	HG-induced SV40 MES 13	HMGB1/TLR4/NF-κB	Promote proliferation, oxidative stress, ECM accumulation, and inflammation in mesangial cells	175
	*In vivo*	HMGB1-induced SV40 MES 13	HMGB1/PTEN/PI3K/Akt	Promote proliferation in mesangial cells	176
	*In vitro, In vivo*	db/db mice	Hspa9/HMGB1	Promote proliferation and fibrosis in mesangial cells	177
	*In vivo*	IFN-γ- induced MMC	JAK2 / STAT1	Promote lipogenesis in mesangial cells	74
	*In vivo*	TWEAK and anti-dsDNA IgG-induced MMC	TWEAK/Fn14; NF‐κB/PI3K/AKT	Promote anti-dsDNA IgG penetration into mesangial cells	75
	*In vitro, In vivo*	MRL/lpr mice	TLR2/4 and RAGE	Promote inflammation	77
	*In vitro, In vivo*	NAFLD + BDCM-induced renal injury	HMGB1/TLR4	Promote mesangial cell activation	78
	*In vivo*	HMGB1-induced MMC	PI3K/Akt	Promote proliferation of mesangial cell	79
	*In vivo*	HG-induced SV40 MES 13	TLR4/NF-κB	Promote ferroptosis in mesangial cells	80
Endothelial cell	*In vivo*	HMGB1-induced HUVECs	/	Promote angiogenesis in Endothelial Cells	84
	*In vitro, In vivo*	MRL/lpr mice	TLR4/MyD88	Induced glomerular endothelial cell injury	86.
	*In vivo*	sera from AAV patients GEnCs	HMGB1/TLR4	Induced glomerular endothelial cell injury	87

HG: high glucose; STZ: Streptozocin; FLCs: free light chains; EMT: epithelial-mesenchymal transition; IMI: imidacloprid; CsA: Cyclosporine A; (mt DNA: mitochondrial DNA; AAs: aristolochic acids; HFD: High fat diet; ADR: adriamycin.

**Table 2:** Clinical studies of HMGB1 in CKD

CKD population	sample size (CKD/HC)	Measurement method of HMGB1	Sample source	Major findings	References
GN	258/49	ELISA	Serum	HMGB1 was expressed in the sera of patients with renal diseases who underwent renal biopsies, especially among those who had vasculitis including ANCA-GN, Henoch-Schonlein purpura nephritis, and IgAN with glomerular crescents.	93
CKD	177/48	ELISA	Serum	HMGB-1 is elevated significantly in CKD patients and correlates with GFR as well as markers of inflammation and malnutrition.	90
CKD	289/61	ELISA	Serum	HMGB1 levels were significantly higher in CKD patients and related to disease stage	91
CKD	20/20	ELISA	Serum	HMGB-1 were independently associated with asymmetric dimethylarginine.	92
LN	50(SLE)/50	ELISA	Serum	Patients with LN had significantly higher serum HMGB1, and correlated positively to the SLE Disease Activity Index.	94
LN	70(SLE)/35	WB and ELISA	Serum	Serum HMGB1 levels are related to SLEDAI scores and proteinuria.	95
LN	69(SLE)/17	WB	Serum And Urine	Serum and urinary levels of HMGB1 were significantly increased in patients with active LN.	96
LN	61(SLE)/14	WB	Urine	HMGB1 is elevated in the urine of patients with active LN, and associated with LN class.	97
LN	44(LN)/16(SLE)	Flow cytometry	Pbmcs And Urine	High frequencies of MP-HMGB1 in urine of LN patients	98
LN	35(LN)/0	WB	Serum	serum levels of HMGB1 were increased in LN, and there was no change after immunosuppressive therapy.	99
ANCA	74(active AAV)/65(active AAV)	ELISA	Plasma	plasma levels of HMGB1 correlated with initial serum creatinine, and estimated glomerular filtration rate.	100
ANCA	25/13	WB	Serum	HMGB1 is significantly increased in AAV with renal involvement.	101
ANCA	51(VAs)/46(HC)	ELISA	Serum	positive correlation between serum HMGB1 levels and Scr, and 24-hour proteinuria	102
CAPD	62/31	ELISA	Serum	HMGB-1 was elevated significantly in CAPD patients and correlated with indicators of inflammation and malnutrition.	136
ESRD	151(HD)/ 102(PD)	ELISA	Serum	Serum level of HMGB1 in patients on HD was higher than PD, and patients with higher HMGB1 had more complications than patients with lower HMGB1, but there was no difference for the survival rate.	138

GN: glomerulonephritis; CKD: chronic kidney disease; LN: lupus nephritis; ANCA: antineutrophil cytoplasmic antibody; CAPD: continuous ambulatory peritoneal dialysis; ESRD: end-stage renal disease; WB: western blot; ELISA: enzyme linked immunosorbent assay

**Table 3:** Therapeutic strategies targeting HMGB1 in CKD

HMGB1 ancts	Experimental models	Mechanism	Effect on CKD	References
Ethyl pyruvate	STZ-induced DKD; CaCl_2_-induced HK-2	Inhibit HMGB1 phosphorylation and release; Inhibit HMGB1/TLR4/NF-κB	Meliorate albuminuria and glomerular injury; prevent AKI-CKD transition	51, 142, 143
Glycyrrhizic acid	STZ-induced DKD; Zucker diabetic fatty rat	Inhibit HMGB1/RAGE/TLR4; Inhibit HMGB1/TLR4/NF-κB	Improve renal injury and inflammatory responses	145, 146
Grape seed proanthocyanidin extract	UUO	Suppress HMGB1/TLR4/p65/TGF-β1	Alleviates renal fibrosis	61
Korea red ginseng	HFD and D-galactose-induced aging-related renal injury	Reduce extracellular HMGB1	Restore aging-related renal injury	121
Bupleurum polysaccharides	STZ-induced DKD	Interrupt HMGB1/TLR4	Reduce renal inflammation, fibrosis, serum creatinine level and urinary albumin excretion rate	149
Dioscin	ADR-induced renal injury; fructose-induced renal damage	Inhibit HMGB1/NF-κB	Reduce renal oxidative stress and inflammation; inhibit renal fibrosis	150, 151,
*Plantago asiatica L*	puromycin aminonucleoside-induced renal injury	Inhibit HMGB1	Suppress inflammation and apoptosis	152
Isomangiferin	db/db mice	Inhibit HMGB1/NLRP3/NF-κB	Inhibit renal inflammation	153
Troxerutin	methotrexate-induced nephrotoxicity	Inhibit HMGB1/RAGE/NF-κB	Inhibit inflammation and apoptosis, and activate of autophagy	154
Ellagic acid	STZ-induced DKD	Inhibit HMGB1/TLR4/NF-кB	Ameliorate oxidative renal injury	155
Anti-HMGB1 antibody	MRL/lpr lupus-prone mice; BXSB mice; cyclosporine-induced nephrotoxicity	suppress HMGB1 translocation from nuclei; Inhibit HMGB1/TLR4	Against albuminuria; attenuate proteinuria, glomerulonephritis, circulating anti-dsDNA and immune complex deposition.	156-158
esRAGE or HMGB1 A Box	STZ-induced DKD	Block the interaction between HMGB1 and its receptors	Reduce albuminuria, glomerular injuries, interstitial fibrosis, and renal inflammation	159
Dapagliflozin	STZ-induced DKD; HG-induced HK-2; high fat diet-induced DKD	Inhibit HMGB1/TLR2/4/NF-κB; Inhibit HMGB1‑RAGE-NF‑κB	Suppress the self-perpetuating cycle of inflammation and diabetic kidney injury	161-163
Empagliflozin	STZ-induced DKD	attenuate renal HMGB1 levels	Alleviate renal inflammation and oxidative stress	160
Simvastatin	BSXSB mice	Reduce the expression of HMGB1 and TLR4	inhibit the autoimmune response	164
Nano selenium and sildenafil	STZ-induced DKD	Inhibit HMGB1/NF-κB	Improve kidney function, and histopathological changes	165

HG: high glucose; STZ: Streptozocin; HFD: High fat diet; ADR: adriamycin; UUO: unilateral ureteral obstruction.
